# Effects of physical activity recommendations on mindset, behavior and perceived health^☆^

**DOI:** 10.1016/j.pmedr.2019.101027

**Published:** 2019-12-09

**Authors:** Octavia H. Zahrt, Alia J. Crum

**Affiliations:** a Department of Organizational Behavior, Stanford Graduate School of Business, Stanford University, Stanford, CA, United States; b Department of Psychology, Stanford University, Stanford, CA, United States

**Keywords:** Physical activity, Exercise, Mindset, Beliefs, Perceptions, Self-efficacy, Recommendations, Guidelines

## Abstract

This research sought to understand if physical activity recommendations––an integral component of many interventions aiming to promote physical activity––may have unexpected effects on individuals’ mindsets (in this case about the adequacy and health consequences of their physical activity) that can strengthen or weaken recommendation effectiveness. Participants were students and staff at a U.S. West Coast private university, recruited between 2016 and 2019. Two experiments with one-week follow-up periods investigated the effects of viewing recommendations that prescribe a lower (vs. higher) amount of physical activity and provide a liberal (vs. stringent) definition of what counts as physical activity on individuals’ mindsets about the adequacy and health consequences of their physical activity, as well as physical activity-related self-efficacy, physical activity behavior, and perceived health. Study 1 (*N* = 157) showed that exposure to low-and-liberal recommendations (vs. high-and-stringent recommendations) caused participants to adopt the mindset that their physical activity was more adequate, which in turn predicted greater engagement in physical activity and perceived health one week later. Study 2 (*N* = 272) showed that regardless of definition of physical activity (liberal vs. stringent), a lower (vs. higher) amount of recommended physical activity led participants to adopt the mindset that their activity was more adequate. This more adaptive mindset predicted greater self-efficacy and engagement in physical activity in the following week, in addition to better perceived health. Rather than inducing complacency, recommendations prescribing a relatively lower (vs. higher) amount of physical activity may be more effective at promoting physical activity and health by inducing adaptive mindsets.

## 1 Introduction

Physical inactivity is a risk factor for heart disease, diabetes and cancer (U.S. Department of Health and Human Services, HHS, 2018). In recent decades, recommendations and guidelines have been developed to inform people about healthy levels of physical activity and motivate behavior change, yet 78% of U.S. adults still fall short of such recommendations (National Center for Health Statistics, NCHS, 2017). There are many reasons for this alarming lack of physical activity, including environmental, social, and psychological factors (Bauman et al., 2012).

This paper focuses on the underappreciated role of psychological mindsets. We posit that individuals hold mindsets about the adequacy of their level of physical activity and its corresponding health consequences (*activity adequacy mindsets*). Individuals adopt these mindsets to reduce uncertainty around what it means to get an adequate amount of physical activity (e.g., how much activity is needed; what activities count as “good exercise”). For any given level of physical activity, individuals may hold the mindset that their activity level is adequate or inadequate, and thus beneficial or harmful to their health.

Prior research suggests that activity adequacy mindsets may influence physical activity behavior and health. First, the mindset that one’s activity level is adequate may boost self-efficacy, which is in part based on past performance accomplishments (Bandura, 1977). Increased self-efficacy in turn promotes engagement in physical activity (Sallis et al., 1988). Second, activity adequacy mindsets may elicit processes similar to placebo effects, that is, responses to treatments caused by individuals’ beliefs and expectations (Petrie and Rief, 2019). The mere belief that one is engaging in exercise accounts for half of the mental health benefits of exercise (Lindheimer et al., 2015). Additionally, the mindset that one’s work is adequate physical activity can improve physiological health in the absence of behavior change (Crum and Langer, 2007). Conversely, patients who expect a treatment to elicit side-effects frequently experience corresponding symptoms and impaired quality of life, even if they are receiving placebo treatments (Petrie and Rief, 2019). Similarly, the mindset that one’s activity level is harmful to one’s health may lead individuals to actually perceive themselves as less healthy. Importantly, perceived health strongly predicts mortality over and above numerous indicators of physical and mental health (Idler and Benyamini, 1997).

Given that activity adequacy mindsets matter for health, it is important to examine their determinants. Initial research suggests that these mindsets are informed not only by individuals’ actual activity levels, but also by how they think their activity levels compare to external standards. For example, people’s perceptions of how active they are compared to peers predict engagement in physical activity one year later (Shakya et al., 2015) and even mortality risk (Zahrt and Crum, 2017), controlling for actual activity levels.

Physical activity recommendations are designed to educate and motivate people to meet healthy activity levels. However, given the growing scientific evidence base and debates about how to distill it into simple recommendations (Warburton and Bredin, 2016), these recommendations have changed over time. The 1996 Surgeon General’s recommendations (CDC, 1996) recommended a relatively low amount of 150 minutes of moderate aerobic activity per week, whereas the official 2018 guidelines (HHS, 2018) additionally require biweekly muscle-strengthening. Moreover, these recommendations differ in whether or not they include light-intensity activities such as leisurely walking in their definition of what counts as healthy activity. Although prior studies manipulating amount or type of recommended activity found null or trivial effects on activity adherence (Rhodes et al., 2009), these characteristics may affect activity adequacy mindsets, sometimes with unintended consequences.

We predict that recommendations prescribing a relatively low amount of physical activity and a liberal definition of what counts as “good activity” will lead individuals to adopt a more positive activity adequacy mindset, compared to recommendations prescribing a higher amount of activity and a more stringent definition (H1). This more positive mindset in turn will predict higher self-efficacy and future engagement in physical activity (H2), and lead individuals to feel healthier (H3). Two experiments test these predictions.

Full details on methods and results, including a validation of the activity adequacy mindset measure, are included in the SOM.

## 2 Study 1

### 2.1 Methods

#### 2.1.1 Participants and procedure

Participants were students and staff at a private U.S. West Coast university (n = 157) attending a one-hour mass-testing session in return for $25 (see Table 1 for sample characteristics).

**Table 1:** Study 1 and 2 baseline characteristics of all participants who completed the baseline survey (Time 1 sample) and the subset of participants who returned for the follow-up survey (Time 2 sub-sample).

Study 1	Time 1 Sample	Time 2 Sub-Sample
**N**	157	137
**Age (Mean, SD)**	23.1 (6.1)	23.2 (6.0)
**Gender (N, Proportion)**		
Female	91 (58%)	83 (62%)
Male	65 (41%)	50 (37%)
Other	1 (1%)	1 (1%)
**Race/ Ethnicity (N, Proportion)**		
Asian/ Asian-American	55 (35%)	47 (35%)
Black/ African-American	8 (5%)	8 (6%)
Hispanic/ Latino/a	11 (7%)	9 (7%)
White/ European-American	57 (36%)	49 (37%)
Other	1 (1%)	0 (0%)
Mixed	25 (16%)	21 (16%)
**University Affiliation (N, Proportion)**		
Student	137 (87%)	116 (87%)
Staff	16 (10%)	14 (10%)
Unknown	4 (3%)	4 (3%)
**Baseline Physical Activity in Hours per Week (Mean, SD)**		
Moderate aerobic activity	5.4 (6.2)	4.6 (4.3)
Vigorous aerobic activity	1.8 (2.8)	1.7 (2.9)
Muscle-strengthening	1.3 (1.7)	1.0 (1.3)

**Study 2**	**Time 1 Sample**	**Time 2 Sub-Sample**

**N**	272	214
**Age (Mean, SD)**	24.1 (7.5)	24.6 (8.1)
**Gender (N, Proportion)**		
Female	173 (64%)	139 (65%)
Male	94 (35%)	73 (34%)
Other	5 (2%)	2 (1%)
**Race/ Ethnicity (N, Proportion)**		
Asian/ Asian-American	101 (37%)	78 (36%)
Black/ African-American	10 (4%)	6 (3%)
Hispanic/ Latino/a	27 (10%)	21 (10%)
White/ European-American	91 (33%)	74 (35%)
Other	5 (2%)	4 (2%)
Mixed	38 (14%)	31 (14%)
**University Affiliation (N, Proportion)**		
Student	227 (83%)	173 (81%)
Staff	39 (14%)	36 (17%)
Other	5 (2%)	4 (2%)
Unknown	1 (0%)	1 (0%)
**Baseline Physical Activity in Hours per Week (Mean, SD)**		
Walking	5.7 (7.7)	5.6 (8.2)
Moderate physical activity	4.5 (5.8)	4.1 (4.7)
Vigorous physical activity	3.9 (4.9)	3.7 (4.6)

*Note*: Participants were affiliates at a U.S. West Coast private university, recruited between 2016 and 2019.

Participants were blinded to the study purpose; they were told that they would answer health-related questions and learn about physical activity guidelines. After providing consent, participants completed the baseline survey via Qualtrics. First, participants reported their baseline physical activity. Next, unbeknownst to them, they were randomly assigned to view one of two versions of physical activity recommendations. The *low-and-liberal* recommendations (based on the 1996 recommendations; CDC, 1996) prescribed ≥150 min of moderate aerobic activity per week and provided a liberal definition of what counts as activity (e.g., jogging, swimming and basketball, but also lighter everyday activities such as walking or housework). The *high-and-stringent* recommendations (based on the 2018 guidelines; HHS, 2018) prescribed a higher amount of activity (≥150 min of moderate or ≥75 min of vigorous aerobic activity, and muscle strengthening ≥2 times per week), and provided a more stringent definition of what counts as activity (e.g., running, swimming, basketball and weight lifting, without lighter everyday activities). Finally, participants completed the mindset measure.

One week later, participants were emailed a link to participate in the follow-up survey measuring the dependent variables (physical activity and perceived health), which 134 participants (85%) completed.

#### 2.1.2 Measures

*Activity adequacy mindset* was assessed using a 7-item measure including items such as “My current level of physical activity is healthy” (7-point scale: Strongly disagree – Strongly agree); Cronbach’s α = 0.92. A validation study demonstrating adequate internal consistency, discriminant validity, convergent validity, and test–retest reliability is included in the SOM. Higher scores denote more positive mindsets. Mindset was assessed immediately post-manipulation.

*Physical activity* was assessed using an adapted version of the International Physical Activity Questionnaire (IPAQ; Craig et al., 2003), immediately pre-manipulation (Time 1) and one week post-manipulation (Time 2).

*Perceived health* was assessed using an item from the CDC HRQOL-14 Healthy Days Measure (Moriarty et al., 2003): “In general, would you say your health is…” (5-point scale: Excellent - Poor). Perceived health was assessed at Time 2.

#### 2.1.3 Analyses

Effects of recommendations on activity adequacy mindset and DVs were analyzed using multiple regression with robust standard errors and mediation analysis with bootstrapped confidence intervals in R (R Core Team, 2019). Standardized coefficients are reported. All analyses controlled for baseline physical activity. Linear regression assumptions were checked and additional analyses confirmed integrity of inferences. Demographics were not predicted to alter recommendations’ effects (which was confirmed through preliminary analyses) and thus not included in final analyses. Differing degrees of freedom reflect response rates at Time 1 and Time 2.

### 2.2 Results

As predicted in H1, regression analysis showed that participants exposed to low-and-liberal (vs. high-and-stringent) recommendations formed more positive *activity adequacy mindsets*, believing that their activity level was more adequate and healthy (*b* = 0.42, *t*(153) = 3.131, *p* = .002).

In support of H2, mediation analysis showed that low-and-liberal (vs. high-and-stringent) recommendations had a positive indirect effect on *physical activity* assessed one week later––mediated by mindset––which trended in the hypothesized direction but did not reach significance at the α = 0.05 level (indirect effect = 0.11, 95% CI [-0.01, 0.23], *p* = .078). There was no direct effect of recommendations on activity (*p* = .896).

As predicted in H3, mediation analysis showed that low-and-liberal (vs. high-and-stringent) recommendations had a positive indirect effect on *perceived health* assessed one week later, mediated by mindset (indirect effect = 0.24, 95% CI [0.08, 0.40], *p* < .001). There was no direct effect of recommendations on perceived health (*p* = .390).

## 3 Study 2

Study 2 was designed to replicate and extend Study 1 by teasing apart effects of the two dimensions manipulated in the recommendations (i.e., amount and definition of what counts as physical activity), and by examining the full process whereby exposure to recommendations predicts subsequent physical activity via mindset and self-efficacy (H2). This study was pre-registered on AsPredicted (available at https://aspredicted.org/bh3st.pdf).

### 3.1 Methods

#### 3.1.1 Participants and procedure

Participants were students and staff at a private U.S. university; 272 completed the baseline survey, 214 (79%) completed the one-week follow-up survey (see Table 1 for sample characteristics). Procedures and information included in the manipulations were equivalent to Study 1, except that a 2 (amount of prescribed activity: low vs. high) X 2 (definition of what counts as activity: liberal vs. stringent) factorial design was used to tease apart the two recommendations dimensions.

#### 3.1.2 Measures

The same measures as in Study 1 were assessed, except that Study 2 used the original validated IPAQ. Additionally, Study 2 measured exercise self-efficacy (Sallis et al., 1988) at Time 2: Participants indicated how confident they were that they could consistently do various behaviors (e.g., “Stick to your exercise program even when you have excessive demands at work/ school”; 5-point scale: Not confident at all - Extremely confident; Cronbach’s α = 0.91).

#### 3.1.3 Analyses

The analytical approach replicated Study 1, with the addition of path analysis with robust standard errors. Our pre-registered predictions regarded the main effects of the two manipulations, so results from additive models are reported. The main effect of the definition manipulation was not statistically significant in any of the models tested (*p’*s ≥ .212). Therefore, results focus on the amount manipulation.

### 3.2 Results

As predicted in H1, regression analysis showed that participants exposed to a low (vs. high) recommended amount of activity formed more positive *activity adequacy mindsets*, believing that their activity level was more adequate and healthy (*b* = 0.25, *t*(268) = 2.162, *p* = .031).

**Fig. 1:**
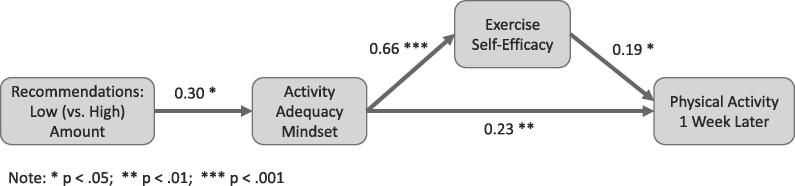
Results from Study 2 path analysis showing the positive indirect effect of low (vs. high) recommended amount of physical activity on physical activity behavior assessed one week later. Note: Participants were affiliates at a U.S. West Coast private university, recruited between 2016 and 2019. *** <0.001; ** <0.01; * <0.05.

In support of H2 and replicating Study 1, mediation analysis showed that low (vs. high) recommended amount of activity had a positive indirect effect on *physical activity* assessed one week later, mediated by mindset (indirect effect = 0.08, 95% CI [0.01, 0.16], *p* = .008). There was no direct effect of recommended amount on activity (*p* = .176). Path analysis was used to test the full process predicted by H2 (Fig. 1). The model fit the data well (χ^2^(6) = 8.465, *p* = .206; RMSEA = 0.041, 90% CI [0.000, 0.100]). Low (vs. high) recommended activity positively affected mindset (*b* = 0.30, *SE* = 0.12, *z =* 2.466, *p* = .014). Mindset in turn positively predicted self-efficacy (*b* = 0.66, *SE =* 0.05, *z* = 13.486, *p* < .001). Finally, mindset and self-efficacy jointly predicted follow-up physical activity (mindset: *b =* 0.23, *SE* = 0.08, *z* = 2.732, *p* = .006; self-efficacy: *b* = 0.19, *SE* = 0.09, *z* = 2.117, *p* = .034).

As predicted in H3, mediation analysis showed that low (vs. high) amount of recommended activity had a positive indirect effect––mediated by mindset––on *perceived health* one week later (0.16, 95% CI [0.04, 0.28], *p* = .004). There was no direct effect of recommendations on perceived health (*p* = .486).

Extending Study 1, these results showed that recommendations’ effects on physical activity and perceived health were driven by recommended amount and provided evidence for the full process including mindset and self-efficacy.

## 4 Discussion

Recommendations are an important tool to encourage people to meet health-promoting activity levels. However, knowledge of recommendations does not predict increased engagement in physical activity (Morrow et al., 2004), and only about one in five Americans meet the current guidelines (NCHS, 2017), which prescribe a relatively high amount of activity (HHS, 2018). This research suggests that paying attention to how these recommendations affect mindsets about the adequacy of one’s activity may be an overlooked piece of the puzzle. Specifically, this study showed that recommendations prescribing a lower amount of activity promote more adaptive activity adequacy mindsets, that is, the mindset that one’s activity level is adequate and beneficial to one’s health. This mindset in turn predicts greater levels of self-efficacy and physical activity one week after briefly viewing these recommendations. Finally, this mindset predicts better perceived health, an important predictor of longevity (Idler and Benyamini, 1997). These findings are in line with recent research showing that mindsets about health-related behaviors such as diet (e.g., Turnwald and Crum, 2019) and physical activity (e.g., Crum and Langer, 2007, Zahrt and Crum, 2017) can have important effects on behavior and health outcomes.

### 4.1 Limitations and future directions

This research relied on samples of relatively active university affiliates, thus future research is needed to establish generalizability. In fact, effects may be stronger in representative samples, as individuals with lower activity levels likely feel more inadequate when exposed to high-amount recommendations or definitions of “good activity” that discount some or all of their sources of physical activity. Additionally, we note that our recommendations manipulations predicted physical activity and perceived health only indirectly through changes in mindset, which we expected given that these outcomes are multiply determined and difficult to change. Finally, physical activity and perceived health were assessed using self-report, the latter using a single item. Although these are standard validated measures (Craig et al., 2003, Idler and Benyamini, 1997, Moriarty et al., 2003), additional research using objective measures over extended follow-up periods is needed.

## 5 Conclusion

This research suggests that a better understanding of mindsets may inform the design of more effective physical activity recommendations. For example, recommendations could encourage individuals to meet optimal amounts of activity while affirming that they can gain substantial health benefits even at lower activity levels (e.g., Wen et al., 2011). Recommendations that promote adaptive mindsets in addition to behavior change are most likely to foster healthy lifestyles and wellbeing.

## CRediT authorship contribution statement

**Octavia H. Zahrt:** Conceptualization, Methodology, Investigation, Formal analysis, Project administration, Software, Validation, Visualization, Writing - original draft, Writing - review & editing. **Alia J. Crum:** Conceptualization, Methodology, Writing - original draft, Writing - review & editing.

## Declaration of Competing Interest

The authors declare that they have no known competing financial interests or personal relationships that could have appeared to influence the work reported in this paper.

## References

[b0005] Bandura A. (1977). Self-efficacy: toward a unifying theory of behavioral change. Psychol. Rev..

[b0010] Bauman A.E., Reis R.S., Sallis J.F., Wells J.C., Loos R.J.F., Martin B.W. (2012). Correlates of physical activity: why are some people physically active and others not?. Lancet.

[b0015] Centers for Disease Control and Prevention (1996). Physical activity and health: a report of the Surgeon General.

[b0020] Craig C.L., Marshall A.L., Sjöström M.M., Bauman A.E., Booth M.L., Ainsworth B.E., Pratt M., Ekelund U., Yngve A., Sallis J.F., Oja P. (2003). International physical activity questionnaire : 12 – country reliability and validity. Med. Sci. Sports Exerc..

[b0025] Crum A.J., Langer E.J. (2007). Mind-set matters: exercise and the placebo effect. Psychol. Sci..

[b0030] Idler E.L., Benyamini Y. (1997). Self-rated health and mortality: a review of twenty-seven community Studies. J. Health Soc. Behav..

[b0035] Lindheimer J.B., O’Connor P.J., Dishman R.K. (2015). Quantifying the placebo effect in psychological outcomes of exercise training: a meta-analysis of randomized trials. Sport. Med..

[b0040] Moriarty D.G., Zack M.M., Kobau R. (2003). Health and quality of life outcomes – the Centers for Disease Control and Prevention’s healthy days measures. Health Qual. Life Outcomes.

[b0045] Morrow J.R., Krzewinski-Malone J.A., Jackson A.W., Bungum T.J., Fitzgerald S.J. (2004). American adults’ knowledge of exercise recommendations. Res. Q. Exerc. Sport.

[b0050] National Center for Health Statistics (2017). Health, United States, 2016: with chartbook on long-term trends in health.

[b0055] Petrie K.J., Rief W. (2019). Psychobiological mechanisms of Placebo and Nocebo effects: pathways to improve treatments and reduce side effects. Annu. Rev. Psychol.

[b0060] R Core Team, 2019. R: A language and environment for statistical computing.

[b0065] Rhodes R.E., Warburton D.E.R., Murray H. (2009). Characteristics of physical activity guidelines and their effect on adherence: a review of randomized trials. Sport. Med..

[b0070] Sallis J.F., Pinski R.B., Grossman R.M., Patterson T.L., Nader P.R. (1988). The development of self-efficacy scales for health-related diet and exercise behaviors. Health Educ. Res..

[b0075] Shakya H.B., Christakis N.A., Fowler J.H. (2015). Self-comparisons as motivators for healthy behavior. Obesity.

[b0080] Turnwald B.P., Crum A.J. (2019). Smart food policy for healthy food labeling: leading with taste, not healthiness, to shift consumption and enjoyment of healthy foods. Prev. Med. (Baltim.).

[b0085] U.S. Department of Health and Human Services (2018). Physical activity guidelines for Americans.

[b0090] Warburton D.E.R., Bredin S.S.D. (2016). Reflections on physical activity and health: what should we recommend?. Can. J. Cardiol..

[b0095] Wen C.P., Wai J.P.M., Tsai M.K., Yang Y.C., Cheng T.Y.D., Lee M.C., Chan H.T., Tsao C.K., Tsai S.P., Wu X. (2011). Minimum amount of physical activity for reduced mortality and extended life expectancy: a prospective cohort study. Lancet.

[b0100] Zahrt O.H., Crum A.J. (2017). Perceived physical activity and mortality: evidence from three nationally representative U.S. samples. Heal. Psychol..

