# Supplementary material for: Effects of physical activity recommendations on mindset, behavior and perceived health
Source: Prev Med Rep. 2019 Dec 9;17:101027. doi: 10.1016/j.pmedr.2019.101027 (PMC6948259; doi:10.1016/j.pmedr.2019.101027)
Supplement: Supplementary data 1 [file mmc1.docx]

­Supplemental Online Materials for

Effects of Physical Activity Recommendations on Mindset, Behavior and Perceived Health

Octavia H. Zahrt

Alia J. Crum

Stanford University

Author Note

Octavia H. Zahrt, Department of Organizational Behavior, Stanford Graduate School of Business, Stanford University; Alia J. Crum, Department of Psychology, Stanford University.

Correspondence concerning this document should be addressed to Octavia Zahrt, Stanford Graduate School of Business, 655 Knight Way, Stanford, CA 94305. E-mail: zahrt@stanford.edu.

Table of Contents

[1. Ethics Statement 2](#_Toc17888735)

[2. Study 1 Supplement 2](#_Toc17888736)

[2.1. Methods 2](#_Toc17888737)

[2.2. Results 5](#_Toc17888740)

[3. Study 2 Supplement 8](#_Toc17888741)

[3.1. Methods 8](#_Toc17888742)

[3.2. Results 10](#_Toc17888744)

[4. Supplemental Figures 15](#_Toc17888745)

[5. Supplemental References 23](#_Toc17888746)

[Appendix 24](#_Toc17888747)

[6. Activity Adequacy Mindset Scale (AAMS) Validation 24](#_Toc17888748)

[7. Pre-Registration Document 31](#_Toc17888756)

# 1. Ethics Statement

The Stanford University institutional review board (IRB) reviewed and approved the protocol for the studies reported here (Protocol # 37424).

# 2. Study 1 Supplement

# 2.1. Methods

**2.1.1. Participants**

Participants were students and staff at a private U.S. university attending one of two one-hour mass-testing sessions in return for $25. In each session, participants completed the baseline survey on a lab computer via the Qualtrics online platform. It was determined in advance that the sample size would be based on however many participants attended either (but not both) of these two sessions. Of the initial 205 recorded responses, 10 were discarded because respondents did not indicate their email address (the only available identifier), such that we could neither determine whether they provided duplicate responses, nor email them the follow-up survey. An additional 31 were duplicate responses (i.e., they stemmed from participants who attended both testing sessions); for each duplicate respondent, their second response was discarded. An additional 7 respondents were excluded as they indicated that their knowledge of English was “fair”, “somewhat poor”, or “extremely poor”. This yielded a sample of 157 participants.

One week after completing the baseline survey including the manipulations, participants were contacted via email to participate in a follow-up survey in return for a $2 Amazon gift card. Of the initial 157 participants, 134 responded to the follow-up survey (85% retention rate). There was no differential attrition by condition as indicated by a two-sample chi-squared test for equality of proportions, χ^2^(1) < .001, *p* = .994. Sample characteristics are given in Table 1 in the main article.

Power analysis showed that in order to detect our predicted indirect effects (H2, H3) with power of 0.80 at the α = .05 level, a sample size of n = 107 was sufficient. Power analysis was conducted following a method introduced by Schoemann, Boulton, & Short (2017), with the following parameters:

- Predicted std. coefficient a –– recommendations (low-and-liberal vs. high-and-stringent) 🡪 AAMS: 0.4
- Predicted std. coefficient b –– AAMS 🡪 physical activity/ perceived health: 0.3
- Predicted std. coefficient c’ –– direct effect recommendations (low-and-liberal vs. high-and-stringent) 🡪 physical activity/ perceived health: 0

**2.1.2. Design and procedure**

After providing their consent, participants were asked to report their physical activity in the last 7 days as a baseline measure of physical activity. Then, they were told that they would now “learn about the public health guidelines for physical activity, issued by the U.S. Department of Health and Human Services.” Next, participants were randomly assigned (between subjects) to view one of two versions of physical activity recommendations. The *low-and-liberal* recommendations prescribed a relatively low amount of physical activity and provided a liberal definition of what counts as physical activity (see Figure S1a). *The high-and-stringent* recommendations prescribed a higher amount of physical activity and provided a more stringent definition of what counts as physical activity (see Figure S1b). After viewing the manipulations, participants completed the activity adequacy mindset scale. One week later, participants were invited to complete the follow-up survey via email, in which they completed the follow-up measures of physical activity and perceived health.

# 2.1.3. Measures

**Activity adequacy mindset.** A 7-item mindset measure designed for this study included the following items and response scales:

- “My current level of physical activity is healthy” (7-point scale: Strongly agree – Strongly disagree)
- “My current level of physical (in-)activity is unhealthy” (7-point scale: Strongly agree – Strongly disagree)
- “My current level of physical activity is helping me achieve or maintain a healthy body weight” (7-point scale: Strongly agree – Strongly disagree)
- How beneficial is your current level of physical activity for your health? (5-point scale: Not at all beneficial – Extremely beneficial)
- How harmful is your current level of physical activity for your health? (5-point scale: Not at all harmful – Extremely harmful)
- How much does your current level of physical (in-)activity increase or decrease your risk of disease? (7-point scale: Increases my risk very much – Decreases my risk very much)
- How much is your current level of physical (in-)activity strengthening or weakening your muscles? (7-point scale: Strengthening very much – Weakening very much)

Scale items were averaged into a composite score after reverse-coding negatively worded items and multiplying 5-point scale items by 1.4 to ensure all items ranged from 1-7. Higher values reflect more positive mindsets. The scale had good internal validity (Cronbach’s α = 0.92). Mindset was assessed in the baseline survey (Time 1, post-manipulation). Details on reliability and validity of the scale are included in the validation study in Section 6 of this supplement.

**Physical activity.** The measure of participants’ aerobic and muscle-strengthening activity was adapted from the International Physical Activity Questionnaire (IPAQ), Short Last 7-Days Self-Administered Format (Craig et al., 2003). To adapt the IPAQ scale for the purpose of our study, we added a measure of muscle-strengthening activity, and we omitted the measures of walking (which we included under moderate aerobic activity) and sitting (which was not relevant to this study). In particular, participants were asked to indicate on how many days in the last 7 days they did “moderate aerobic activity (e.g., walking, biking at regular pace, volleyball)”, and how much time (in minutes) they usually spent doing moderate aerobic activities on one of those days. They were then asked the same questions about their “vigorous aerobic activity (e.g., running, swimming laps, fast bicycling)” and “muscle­strengthening activity (e.g., weight lifting, sit ups, yoga)”.

Adapting the IPAQ scoring protocol (2005), we calculated the summary score for aerobic activity as

*Total aerobic physical activity MET-minutes/week = 4 * moderate activity minutes * moderate days + 8 * vigorous activity minutes * vigorous days.*

Separately, we calculated the summary score for muscle-strengthening activity as

*Total muscle-strengthening minutes/week =* *muscle-strengthening minutes * muscle-strengthening day*s.

Baseline physical activity was assessed at Time 1 (pre-manipulation). Follow-up physical activity was assessed at Time 2 (one week post-manipulation).

**Perceived health.** An item from the CDC HRQOL–14 Healthy Days Measure (CDC, 1993) was used: “In general, would you say your health is…” (5-point scale: Excellent, Very Good, Good, Fair, Poor). Perceived health was assessed at Time 2.

**2.1.4. Analyses**

All measures were standardized prior to analysis as measurement units differed. Effects of manipulations on DVs were analyzed using ordinary least squares (OLS) multiple linear regression and mediation analysis in the statistical software R (Version 3.6.1; R Core Team, 2019). Mediation analyses with bootstrapped confidence intervals (1,000 simulations) were conducted using the ‘mediation’ R package (Tingley, Yamamoto, Hirose, & Keele, 2013). All analyses controlled for baseline physical activity (i.e., for the aerobic activity and muscle-strengthening activity summary scores), given that (a) we aimed to show that manipulations affected mindset independently of actual physical activity, and (b) baseline activity was expected to influence all other measures.

Assumptions underlying linear regression were checked for all models. Diagnostics tests revealed violations of the assumption of homoscedasticity of residuals in most models. Therefore, linear models with robust standard errors were conducted using the ‘estimatr’ R package (Blair et al., 2019). Additionally, in models predicting physical activity, the assumption of normally distributed residuals was violated. This is not necessarily problematic, as departures from normality compromise inferences from linear regression only if the sample is quite small (Weisberg, 2005; Williams, Grajales, & Kurkiewicz, 2013). However, to ensure the integrity of inferences, a bootstrap approach (e.g., Efron & Tibshirani, 1994) was used to replicate findings from linear regression (see Weisberg, 2005) using the R package ‘boot’. In particular, each regression model predicting physical activity was fit 5,000 times on a random sample (with replacement) of the data. The resulting parameter estimates were used to derive bootstrapped coefficient estimates and construct 95% confidence intervals, which were then compared with those derived from the original OLS linear regression models to demonstrate consistency.

# 2.2. Results

Complete results from multiple regression and mediation analyses conducted in Study 1 are reported below.

| Predictors of Activity Adequacy Mindset (Study 1) | | | | |
| --- | --- | --- | --- | --- |
|  | | | | |
|  | Estimate | SE | t-value | p-value |
|  | | | | |
| (Intercept) | -0.22 | 0.11 | -2.097 | .038 |
| Recommended activity (low-and-liberal) | 0.42 | 0.13 | 3.131 | .002 |
| Baseline aerobic physical activity | 0.16 | 0.17 | 0.940 | .349 |
| Baseline muscle-strengthening activity | 0.42 | 0.09 | 4.678 | < 0.001 |
|  |  |  |  |  |

*Note:* 153 degrees of freedom. Multiple R^2^ = 0.32.

| **Predictors of Physical Activity** at Time 2 **(**Study 1**)** | | | | | |
| --- | --- | --- | --- | --- | --- |
|  | | | | | |
|  | Estimate | SE | t-value | p-value |  |
|  | | | | | |
| (Intercept) | -0.04 | 0.10 | - 0.454 | 0.651 |  |
| Recommended activity (low-and-liberal) | 0.08 | 0.14 | 0.589 | 0.557 |  |
| Baseline aerobic physical activity | 0.55 | 0.18 | 2.994 | 0.003 |  |
| Baseline muscle-strengthening activity | 0.01 | 0.11 | 0.047 | 0.963 |  |
|  |  |  |  |  |  |

*Note:* 130 degrees of freedom. Multiple R^2^ = 0.31.

| Confidence Intervals for Predictors of Physical Activity at Time 2 Based on OLS Linear Regression and Bootstrap (Study 1) | | | | | | |
| --- | --- | --- | --- | --- | --- | --- |
|  | | | | | | |
|  | OLS linear regression | | | Bootstrap | | |
|  | Estimate | 2.5 % | 97.5 % | Estimate | 2.5 % | 97.5 % |
|  | | | | | | |
| (Intercept) | -0.04 | -0.23 | 0.15 | -0.06 | -0.23 | 0.12 |
| Recommended activity (low-and-liberal) | 0.08 | -0.20 | 0.37 | 0.10 | -0.15 | 0.41 |
| Baseline aerobic physical activity | 0.55 | 0.19 | 0.92 | 0.50 | 0.27 | 0.89 |
| Baseline muscle-strengthening activity | 0.01 | -0.22 | 0.23 | -0.02 | -0.24 | 0.18 |
|  |  |  |  |  |  |  |

*Note:* Bootstrap with 5,000 iterations.

|  | | | | |
| --- | --- | --- | --- | --- |
| Predictors of Physical Activity at Time 2 (Including **Activity Adequacy** Mindset) **(**Study 1**)** | | | | |
|  | | | | |
|  | Estimate | SE | t-value | p-value |
|  | | | | |
| (Intercept) | 0.01 | 0.09 | 0.107 | .915 |
| Activity adequacy mindset | 0.22 | 0.11 | 2.018 | .046 |
| Recommended activity (low-and-liberal) | -0.02 | 0.14 | -0.162 | .872 |
| Baseline aerobic physical activity | 0.52 | 0.17 | 3.063 | .003 |
| Baseline muscle-strengthening activity | -0.09 | 0.13 | -0.669 | .505 |
|  |  |  |  |  |

*Note:* 129 degrees of freedom. Multiple R^2^ = 0.34.

| Confidence Intervals for Predictors of Physical Activity (Including **Activity Adequacy** Mindset) at Time 2 Based on OLS Linear Regression and Bootstrap (Study 1) | | | | | | |
| --- | --- | --- | --- | --- | --- | --- |
|  | | | | | | |
|  | OLS linear regression | | | Bootstrap | | |
|  | Estimate | 2.5 % | 97.5 % | Estimate | 2.5 % | 97.5 % |
|  | | | | | | |
| (Intercept) | 0.01 | -0.17 | 0.19 | +0.00 | -0.16 | 0.18 |
| Activity adequacy mindset | 0.22 | +0.00 | 0.43 | 0.25 | 0.04 | 0.48 |
| Recommended activity (low-and-liberal) | -0.02 | -0.30 | 0.25 | -0.03 | -0.28 | 0.26 |
| Baseline aerobic physical activity | 0.52 | 0.19 | 0.86 | 0.47 | 0.26 | 0.87 |
| Baseline muscle-strengthening activity | -0.09 | -0.35 | 0.17 | -0.13 | -0.35 | 0.14 |
|  |  |  |  |  |  |  |

*Note:* Bootstrap with 5,000 iterations.

Indirect Effect of Recommendations (Low-and-Liberal vs. High-and-Stringent) on Physical Activity at **Time 2, Mediated** by **Activity Adequacy** Mindset (Covariates: Baseline Physical Activity) **(**Study 1**)**

|  | Estimate | 95% CI | p-value |  |
| --- | --- | --- | --- | --- |
| Indirect effect | 0.11 | [-0.01, 0.23] | .078 |  |
| Direct effect | -0.02 | [-0.26, 0.22] | .896 |  |
| Total effect | 0.08 | [-0.17, 0.33] | .652 |  |
|  |  |  |  |  |

| Predictors of Perceived Health **(**Study 1**)** | | | | |
| --- | --- | --- | --- | --- |
|  | | | | |
|  | Estimate | SE | t-value | p-value |
|  | | | | |
| (Intercept) | -0.06 | 0.11 | -0.536 | .593 |
| Recommended activity (low-and-liberal) | 0.12 | 0.16 | 0.763 | .447 |
| Baseline aerobic physical activity | 0.03 | 0.13 | 0.259 | .796 |
| Baseline muscle-strengthening activity | 0.32 | 0.11 | 3.020 | .003 |
|  |  |  |  |  |

*Note:* 130 degrees of freedom. Multiple R^2^ = 0.12.

| **Predictors of** Perceived **Health (Including Activity Adequacy Mindset) (**Study 1**)** | | | | |
| --- | --- | --- | --- | --- |
|  | | | | |
|  | Estimate | SE | t-value | p-value |
|  | | | | |
| (Intercept) | 0.07 | 0.10 | 0.710 | .479 |
| Activity adequacy mindset | 0.52 | 0.09 | 6.162 | < .001 |
| Recommended activity (low-and-liberal) | -0.13 | 0.15 | -0.867 | .388 |
| Baseline aerobic physical activity | -0.04 | 0.09 | -0.405 | .686 |
| Baseline muscle-strengthening activity | 0.10 | 0.09 | 1.063 | .290 |
|  |  |  |  |  |

*Note:* 129 degrees of freedom. Multiple R^2^ = 0.30.

| Indirect Effect of Recommendations (Low-and-Liberal vs. High-and-Stringent) on Perceived **Health, Mediated** by **Activity Adequacy** Mindset (Covariates: Baseline Physical Activity) **(**Study 1**)** | | | | |  |
| --- | --- | --- | --- | --- | --- |
|  | | | | |  |
|  | Estimate | 95% CI | p-value |  |  |
| Indirect effect | 0.24 | [0.08, 0.40] | < .001 |  |  |
| Direct effect | -0.12 | [-0.38, 0.16] | .390 |  |  |
| Total effect | 0.11 | [-0.17, 0.39] | .410 |  |  |
|  |  |  |  |  | |

# 3. Study 2 Supplement

Study 2 was pre-registered on AsPredicted, including all measures, analyses, and exclusion criteria. The pre-registration is available at https://aspredicted.org/bh3st.pdf.

# 3.1. Methods

**3.1.1. Participants**

Participants were students and staff at a private U.S. university attending one of three one-hour mass-testing sessions in return for $25. It was determined in advance that the sample size would be at least 200 to allow for sufficient statistical power. Of the initial 306 recorded responses, 27 were discarded because respondents did not indicate their email address (the only available identifier), such that we could neither determine whether they provided duplicate responses, nor email them the follow-up survey. An additional 21 were duplicate responses (i.e., they stemmed from participants who attended more than one mass testing sessions); for each duplicate respondent, their second response was discarded. An additional 14 participants were excluded as they had already participated in Study 1. Finally, an additional 6 respondents were excluded as they indicated that their knowledge of English was “fair”, “somewhat poor”, or “extremely poor”. This yielded a sample of 272 participants.

One week after completing the baseline survey including the manipulations, participants were contacted via email to participate in a follow-up survey in return for a $2 Amazon Gift Card. Of the initial 272 participants, 218 responded to the follow-up survey, among whom 214 agreed to have their data used after debriefing (79% retention rate). There was no differential attrition by condition as indicated by a two-sample chi-squared test for equality of proportions, χ^2^(3) = .742, *p* = .863. Sample characteristics are given in Table 1 in the main article.

Power analysis showed that in order to detect our predicted serial mediation effect (H2) with power of 0.80 at the α = .05 level, a sample size of n = 200 was sufficient. Power analysis was based on the following parameters:

- Predicted std. coefficient a1 –– recommended amount (low vs. high) 🡪 AAMS: 0.2
- Predicted std. coefficient a2 –– recommended amount (low vs. high) 🡪 self-efficacy: 0
- Predicted std. coefficient b1 –– AAMS 🡪 physical activity: 0.1
- Predicted std. coefficient b2 –– self-efficacy 🡪 physical activity: 0.3
- Predicted std. coefficient c’ –– direct effect of recommended amount (low vs. high) 🡪 physical activity: 0
- Predicted std. coefficient d –– AAMS 🡪 self-efficacy: 0.6

**3.1.2. Design and procedure**

A 2 (amount of prescribed physical activity: low vs. high) X 2 (definition of what counts as physical activity: liberal vs. stringent) factorial design was used. Procedures were equivalent to Study 1. Information included in the manipulations was also equivalent to Study 1, except that the amount of prescribed physical activity (low vs. high) and the definition of what counts as physical activity (liberal vs. stringent) were separately manipulated (see Figures S2a-S2d for manipulation materials).

# 3.1.3. Measures

**Activity adequacy mindset.** See Study 1.

**Physical activity.** Study 2 used the original validated International Physical Activity Questionnaire (IPAQ), Short Last 7-Days Self-Administered Format (Craig et al., 2003), rather than an adapted version as in Study 1. In particular, participants were asked to indicate on how many days in the last 7 days they did “vigorous activities” (“Vigorous physical activities refer to activities that take hard physical effort and make you breathe much harder than normal.  Think only about those physical activities that you did for at least 10 minutes at a time […] like heavy lifting, digging, aerobics, or fast bicycling”. Participants were then asked how much time (in hours and minutes) they usually spent doing vigorous activities on one of those days. Next, participants were asked the same questions about their “moderate activities” (“Moderate activities refer to activities that take moderate physical effort and make you breathe somewhat harder than normal.  Think only about those physical activities that you did for at least 10 minutes at a time […] like carrying light loads, bicycling at a regular pace, or doubles tennis”). Finally, participants were asked the same questions about walking (“This includes at work and at home, walking to travel from place to place, and any other walking that you have done solely for recreation, sport, exercise, or leisure”).

Following the IPAQ scoring protocol (2005), we calculated the summary score for aerobic activity as

*Total aerobic physical activity MET-minutes/week = 3.3 * walking minutes * walking days + 4 * moderate activity minutes * moderate days + 8 * vigorous activity minutes * vigorous days.*

Baseline physical activity was assessed at Time 1 (pre-manipulation). Follow-up physical activity was assessed at Time 2 (one week post-manipulation).

**Perceived health.** See Study 1.

**Exercise self-efficacy.** Study 2 also assessed self-efficacy for exercise behaviors (Sallis, Pinski, Grossman, Patterson, & Nader, 1988). Out of the original 12 items, we used 8 items in order to keep survey length reasonable and focus only on items most relevant to our study population. In particular, participants indicated how confident they were that they could motivate themselves for at least six months to consistently do the following behaviors:

- Get up early, even on weekends, to exercise.
- Stick to your exercise program after a long, tiring day at work/ school.
- Exercise even though you are feeling stressed or depressed.
- Set aside time for a physical activity program; that is, walking, jogging, swimming, biking, or other continuous activities for at least 30 minutes, 3 times per week.
- Stick to your exercise program when your family is demanding more time from you.
- Stick to your exercise program when you have household chores to attend to.
- Stick to your exercise program even when you have excessive demands at work/ school.
- Stick to your exercise program when social activities and obligations are very time consuming.

Participants responded on a 5-point scale: Not confident at all – Extremely confident, with an additional option to indicate “Does not apply to me”. The scale had good internal validity (Cronbach’s α = .91). Exercise self-efficacy was assessed at Time 2 in the follow-up survey.

**3.1.4. Analyses**

The analytical strategy replicated Study 1. In addition, path analysis using maximum likelihood estimation with robust (Huber-White) standard errors and a scaled test statistic that is (asymptotically) equal to the Yuan-Bentler test statistic was conducted in the ‘lavaan’ R package (Rosseel, 2012).

# 3.2. Results

Complete results from multiple regression analyses, mediation analyses and path analysis in Study 2 are reported below.

| Predictors of Activity Adequacy Mindset **(**Study 2**)** | | | | |
| --- | --- | --- | --- | --- |
|  | | | | |
|  | Estimate | SE | t-value | p-value |
|  | | | | |
| (Intercept) | -0.16 | 0.10 | -1.674 | .095 |
| Recommended amount (low) | 0.25 | 0.11 | 2.162 | .031 |
| Recommended definition (liberal) | 0.08 | 0.12 | 0.665 | .507 |
| Baseline physical activity | 0.34 | 0.09 | 3.777 | < .001 |
|  |  |  |  |  |

*Note:* 268 degrees of freedom. Multiple R^2^ = 0.13.

| **Predictors of Self-Efficacy (**Study 2**)** | | | | |
| --- | --- | --- | --- | --- |
|  | | | | |
|  | Estimate | SE | t-value | p-value |
|  | | | | |
| (Intercept) | -0.16 | 0.12 | -1.418 | .158 |
| Recommended amount (low) | 0.17 | 0.13 | 1.320 | .188 |
| Recommended definition (liberal) | 0.15 | 0.13 | 1.193 | .234 |
| Baseline physical activity | 0.38 | 0.12 | 3.133 | .002 |
|  |  |  |  |  |

*Note:* 210 degrees of freedom. Multiple R^2^ = 0.16.

| **Predictors of Self-Efficacy (Including Activity Adequacy Mindset) (**Study 2**)** | | | | |
| --- | --- | --- | --- | --- |
|  | | | | |
|  | Estimate | SE | t-value | p-value |
|  | | | | |
| (Intercept) | -0.00 | 0.10 | -0.018 | .985 |
| Activity adequacy mindset | 0.61 | 0.05 | 11.351 | < .001 |
| Recommended amount (low) | -0.05 | 0.10 | -0.468 | .640 |
| Recommended definition (liberal) | 0.05 | 0.10 | 0.496 | .621 |
| Baseline physical activity | 0.20 | 0.08 | 2.397 | .017 |
|  |  |  |  |  |

*Note:* 209 degrees of freedom. Multiple R^2^ = 0.48.

| Predictors of Physical Activity at **Time 2** **(**Study 2**)** | | | | |
| --- | --- | --- | --- | --- |
|  | | | | |
|  | Estimate | SE | t-value | p-value |
|  | | | | |
| (Intercept) | 0.04 | 0.11 | 0.369 | .712 |
| Recommended amount (low) | -0.09 | 0.11 | -0.830 | .408 |
| Recommended definition (liberal) | 0.01 | 0.10 | 0.144 | .886 |
| Baseline physical activity | 0.60 | 0.20 | 2.923 | .004 |
|  |  |  |  |  |

*Note:* 210 degrees of freedom. Multiple R^2^ = 0.35.

| Confidence Intervals for Predictors of Physical Activity at Time 2 Based on OLS Linear Regression and Bootstrap (Study 2) | | | | | | |
| --- | --- | --- | --- | --- | --- | --- |
|  | | | | | | |
|  | OLS linear regression | | | Bootstrap | | |
|  | Estimate | 2.5 % | 97.5 % | Estimate | 2.5 % | 97.5 % |
|  | | | | | | |
| (Intercept) | 0.04 | -0.17 | 0.25 | 0.04 | -0.14 | 0.28 |
| Recommended amount (low) | -0.09 | -0.31 | 0.12 | -0.10 | -0.32 | 0.10 |
| Recommended definition (liberal) | 0.01 | -0.19 | 0.22 | 0.02 | -0.18 | 0.22 |
| Baseline physical activity | 0.60 | 0.19 | 1.00 | 0.57 | 0.22 | 0.88 |
|  |  |  |  |  |  |  |

*Note:* Bootstrap with 5,000 iterations.

Predictors of Physical Activity at **Time 2** (Including **Activity Adequacy** Mindset) **(**Study 2**)**

|  | Estimate | SE | t-value | p-value |
| --- | --- | --- | --- | --- |
|  | | | | |
| (Intercept) | 0.10 | 0.10 | 0.939 | .349 |
| Activity adequacy mindset | 0.22 | 0.08 | 2.860 | .005 |
| Recommended amount (low) | -0.17 | 0.12 | -1.408 | .161 |
| Recommended definition (liberal) | -0.02 | 0.10 | -0.209 | .835 |
| Baseline physical activity | 0.53 | 0.21 | 2.528 | .012 |
|  |  |  |  |  |

*Note:* 209 degrees of freedom. Multiple R^2^ = 0.40.

| Confidence Intervals for Predictors of Physical Activity (Including Activity Adequacy Mindset) at Time 2 Based on OLS Linear Regression and Bootstrap (Study 2) | | | | | | |
| --- | --- | --- | --- | --- | --- | --- |
|  | | | | | | |
|  | OLS linear regression | | | Bootstrap | | |
|  | Estimate | 2.5 % | 97.5 % | Estimate | 2.5 % | 97.5 % |
|  | | | | | | |
| (Intercept) | 0.10 | -0.11 | 0.30 | 0.10 | -0.07 | 0.36 |
| Activity adequacy mindset | 0.22 | 0.07 | 0.37 | 0.23 | 0.09 | 0.37 |
| Recommended amount (low) | -0.17 | -0.40 | 0.07 | -0.18 | -0.44 | 0.03 |
| Recommended definition (liberal) | -0.02 | -0.22 | 0.18 | -0.02 | -0.23 | 0.17 |
| Baseline physical activity | 0.53 | 0.12 | 0.94 | 0.50 | 0.17 | 0.83 |
|  |  |  |  |  |  |  |

*Note:* Bootstrap with 5,000 iterations.

Indirect Effect of Recommended Amount (Low vs. High) on Physical Activity at **Time 2, Mediated** by **Activity Adequacy** Mindset (Covariates: Baseline Physical Activity and Recommended Definition) **(**Study 2**)**

|  | Estimate | 95% CI | p-value |
| --- | --- | --- | --- |
| Indirect effect | 0.08 | [0.01, 0.16] | .008 |
| Direct effect | -0.16 | [-0.39, 0.07] | .176 |
| Total effect | -0.09 | [-0.29, 0.13] | .476 |

| **Results of Path Analysis Showing the Full Process Linking Recommended Amount (Low vs. High) with Physical Activity (PA) at Time 2 via Activity Adequacy Mindset and Self-Efficacy (**Study 2**)** | | | | | | | | | |  |
| --- | --- | --- | --- | --- | --- | --- | --- | --- | --- | --- |
|  | | | | | | | | | |  |
| Model Parameter | Estimate | SE | z-value | p-value | |  |  |  |  |  |
| Regressions |  |  |  |  | |  |  |  |  |  |
| Recommended amount (low) → Mindset | 0.30 | 0.12 | 2.466 | .014 | |  |  |  |  |  |
| Recommended definition (liberal) → Mindset | -0.16 | 0.13 | -1.248 | .212 | |  |  |  |  |  |
| Mindset → Self-efficacy | 0.66 | 0.05 | 13.486 | < .001 | |  |  |  |  |  |
| Self-efficacy → Time 2 PA | 0.19 | 0.09 | 2.117 | .034 | |  |  |  |  |  |
| Mindset → Time 2 PA | 0.23 | 0.08 | 2.732 | .006 | |  |  |  |  |  |
| Covariances |  |  |  |  | |  |  |  |  |  |
| *cov*(Time 1 PA, Mindset) | 0.29 | 0.06 | 4.449 | < .001 | |  |  |  |  |  |
| *cov*(Time 1 PA, Self-efficacy) | 0.18 | 0.05 | 3.414 | .001 | |  |  |  |  |  |
| *cov*(Time 1 PA, Time 2 PA) | 0.44 | 0.17 | 2.572 | .010 | |  |  |  |  |  |
|  |  |  |  |  |  |  |  |  |  | |

*Note:* χ^2^(6) = 8.465, *p* = .206; CFI= 0.991; TLI = 0.980; RMSEA = 0.041, 90% CI [0.000, 0.100].

| Predictors of Perceived Health **(**Study 2**)** | | | | |
| --- | --- | --- | --- | --- |
|  | | | | |
|  | Estimate | SE | t-value | p-value |
|  | | | | |
| (Intercept) | -0.06 | 0.12 | -0.463 | .644 |
| Recommended amount (low) | 0.11 | 0.13 | 0.837 | .403 |
| Recommended definition (liberal) | -0.00 | 0.13 | -0.005 | .996 |
| Baseline physical activity | 0.29 | 0.07 | 4.320 | <.001 |
|  |  |  |  |  |

*Note:* 210 degrees of freedom. Multiple R^2^ = 0.09.

| **Predictors of** Perceived **Health (Including** Activity Adequacy **Mindset) (**Study 2) | | | | |
| --- | --- | --- | --- | --- |
|  | | | | |
|  | Estimate | SE | t-value | p-value |
|  | | | | |
| (Intercept) | 0.09 | 0.10 | 0.923 | .357 |
| Activity adequacy mindset | 0.54 | 0.07 | 7.899 | < .001 |
| Recommended amount (low) | -0.08 | 0.12 | -0.709 | .479 |
| Recommended definition (liberal) | -0.09 | 0.11 | -0.779 | .437 |
| Baseline physical activity | 0.13 | 0.06 | 2.224 | .027 |
|  |  |  |  |  |

*Note:* 209 degrees of freedom. Multiple R^2^ = 0.34.

Indirect Effect of Recommended Amount (Low vs. High) on Perceived Health**, Mediated** by **Activity Adequacy** Mindset (Covariates: Baseline Physical Activity and Recommended Definition) (Study 2)

|  | Estimate | 95% CI | p-value |
| --- | --- | --- | --- |
| Indirect effect | 0.16 | [0.04, 0.28] | .004 |
| Direct effect | -0.06 | [-0.25, 0.13] | .486 |
| Total effect | 0.08 | [-0.11, 0.31] | .366 |

# 4. Supplemental Figures

**Figure S1a.** Study 1 recommendations manipulation, low-and-liberal condition.

**
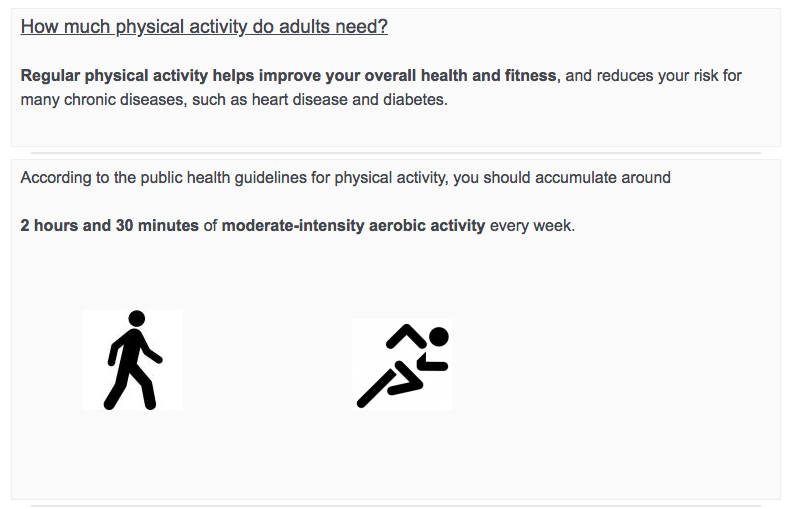
**

**
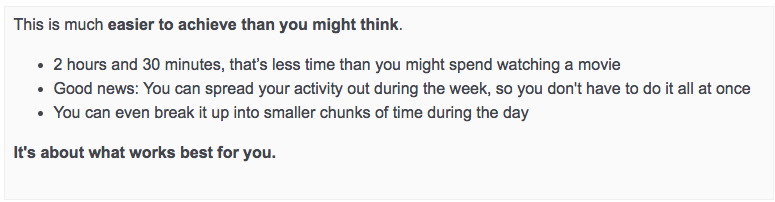
**

**
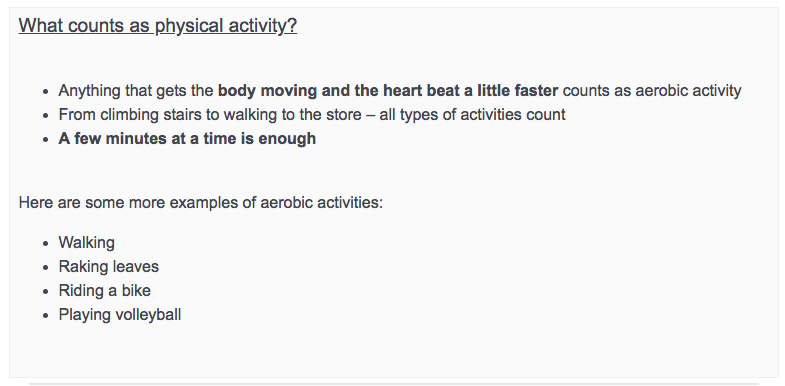
**

**
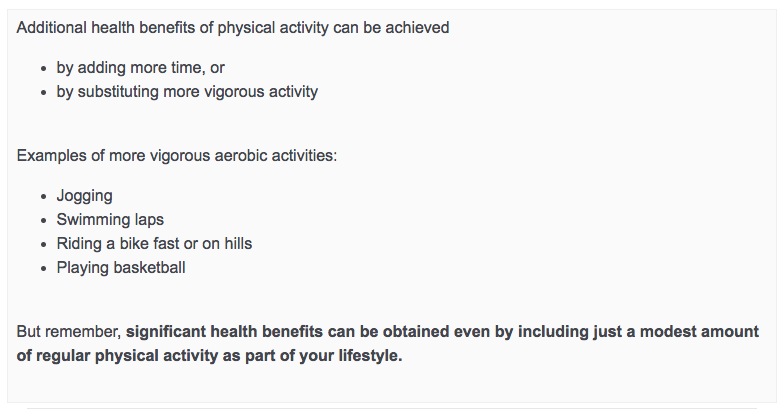
**

**Figure S1b.** Study 1 recommendations manipulation, high-and-stringent condition.

**
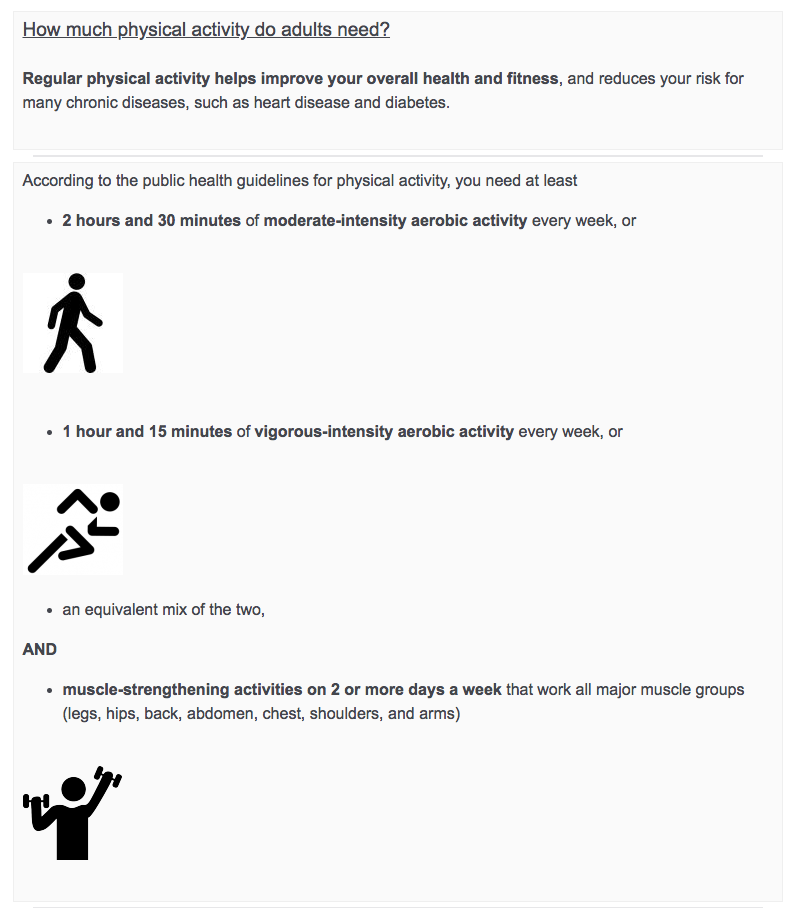
**


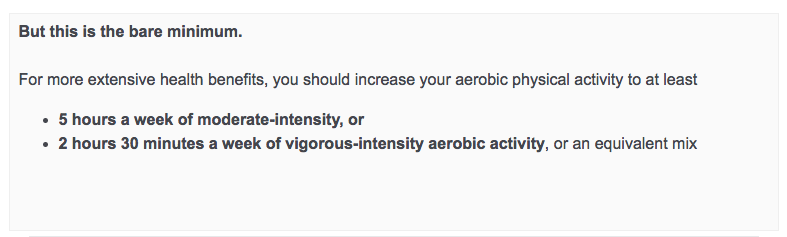


**
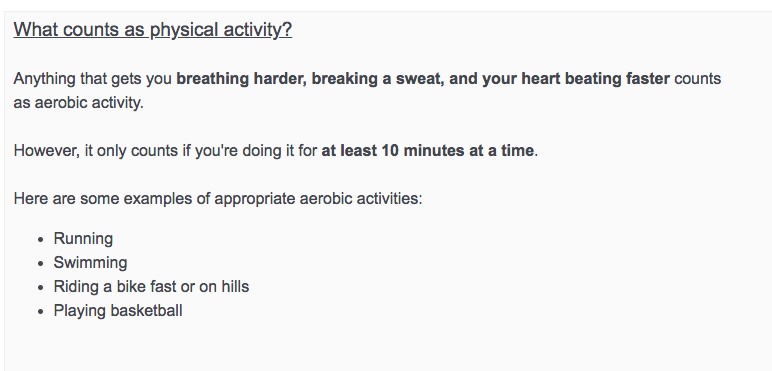
**

**
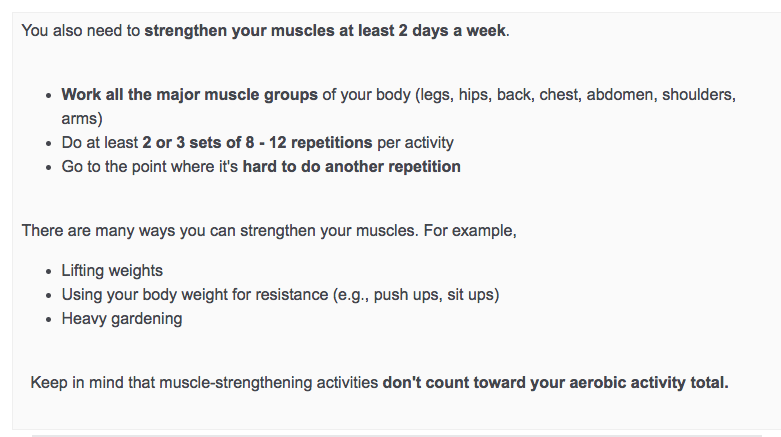
**

**Figure S2a.** Study 2 recommendations manipulation, low-amount condition.


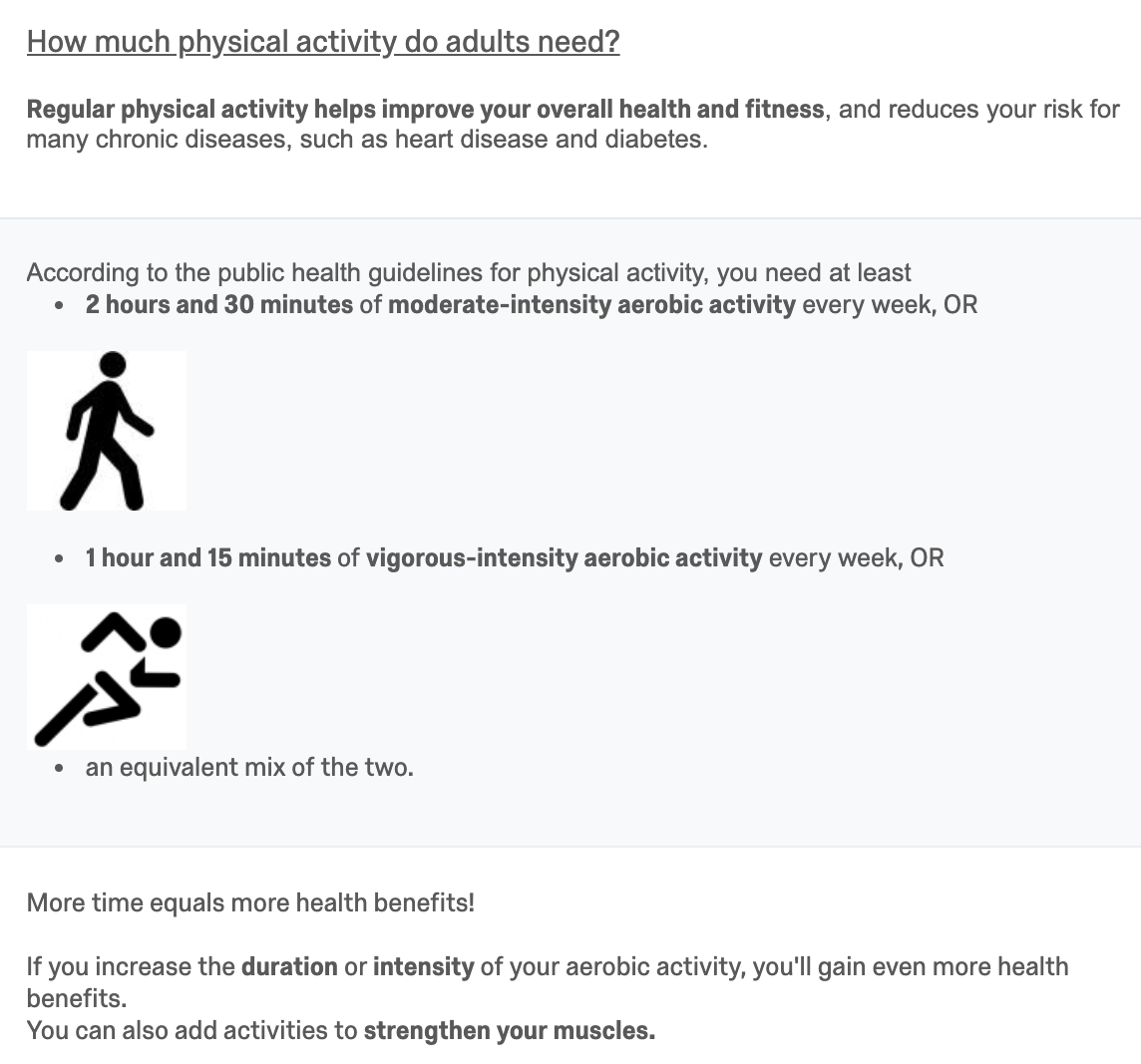


**Figure S2b.** Study 2 recommendations manipulation, high-amount condition.


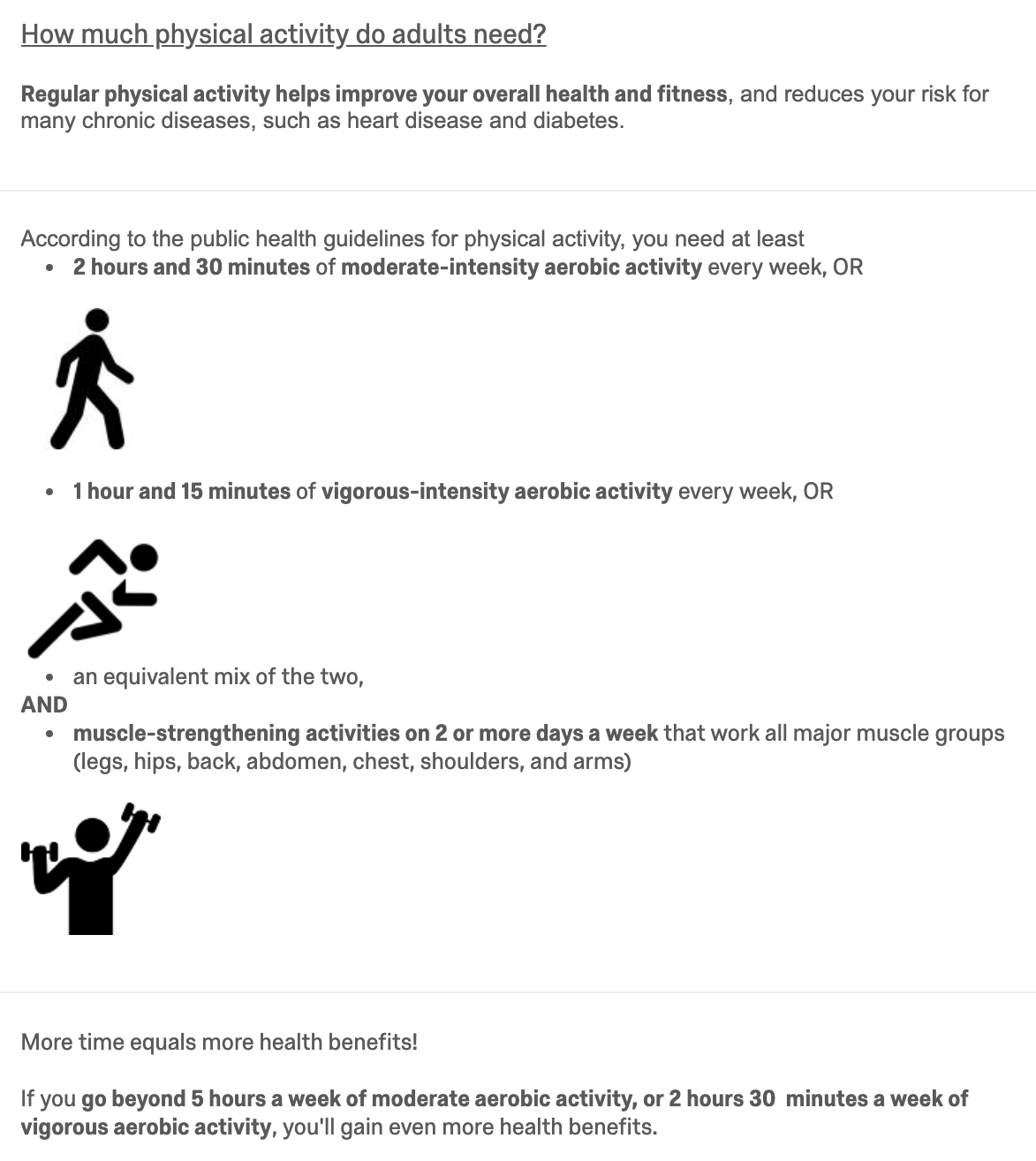


**Figure S2c.** Study 2 recommendations manipulation, liberal-definition condition.


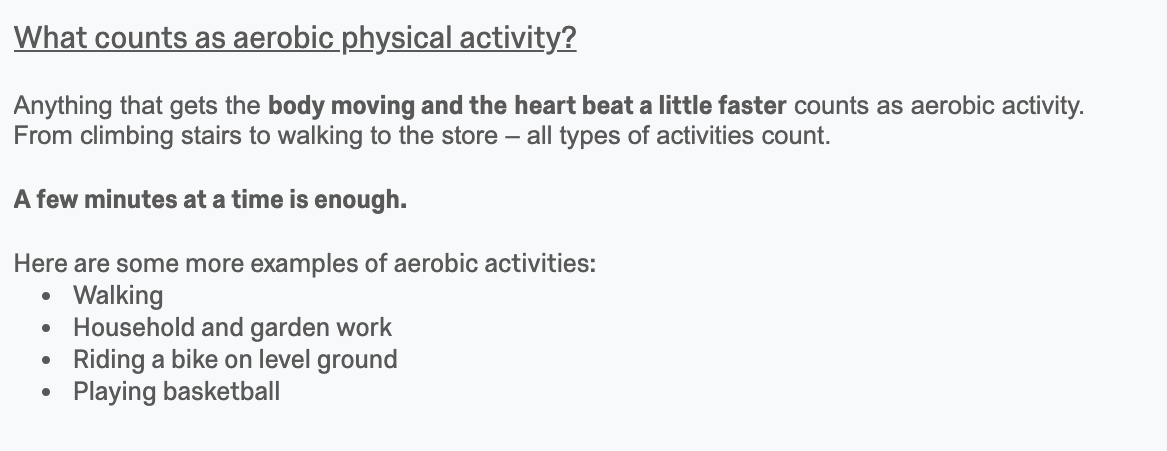

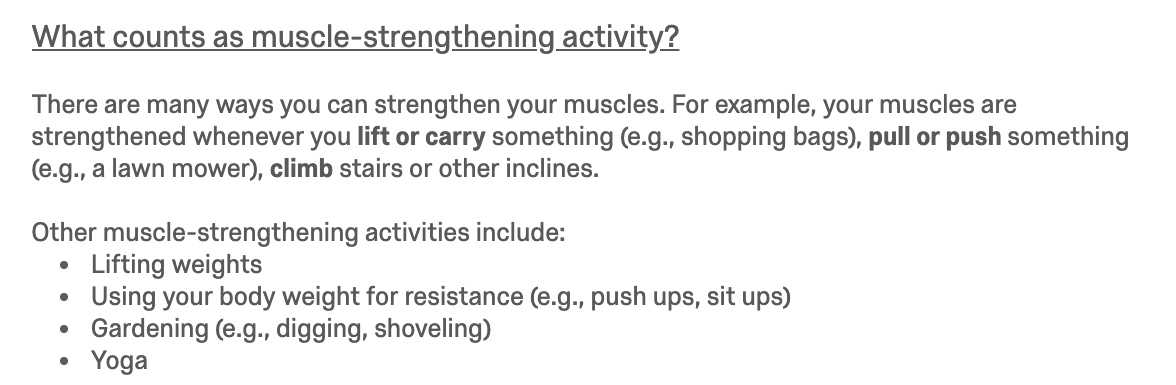


**Figure S2d.** Study 2 recommendations manipulation, stringent-definition condition.


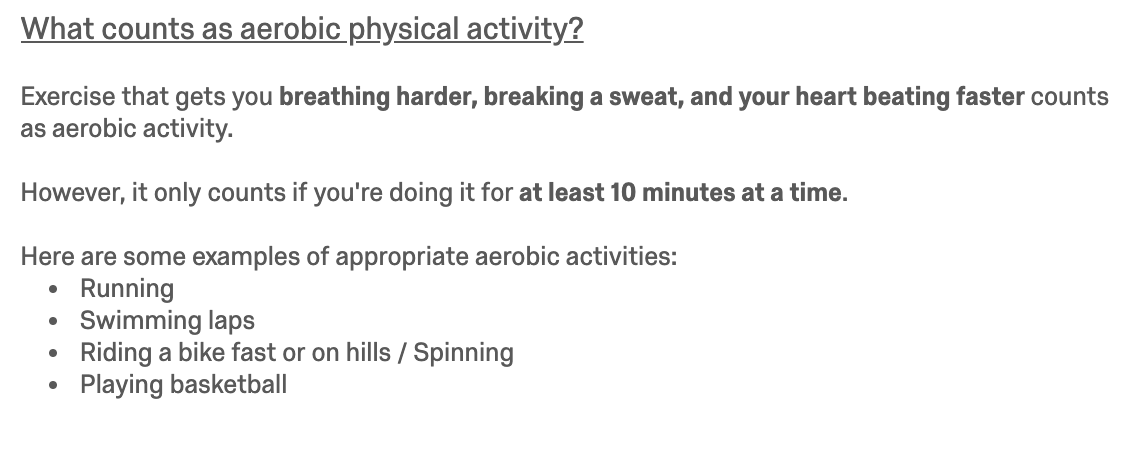


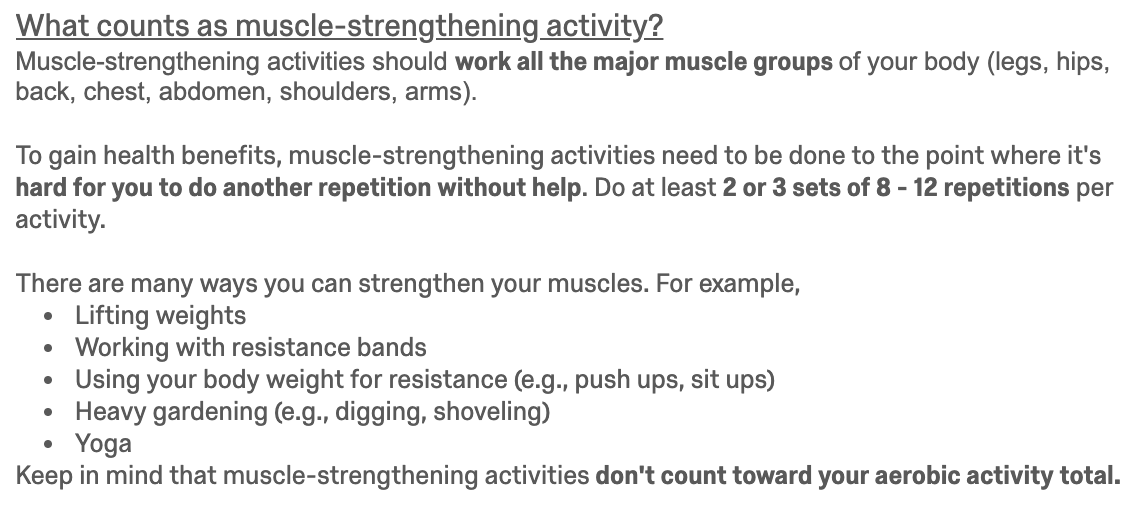


# 5. Supplemental References

Blair, G., Cooper, J., Coppock, A., Humphreys, M., Sonnet, L., Fultz, N., & Medina, L. (2019). *Fast Estimators for Design-Based Inference.*

Cohen, S., Kamarck, T., & Mermelstein, R. (1983). A Global Measure of Perceived Stress. *Journal of Health and Social Behavior*, *24*(4), 385. https://doi.org/10.2307/2136404

Craig, C. L., Marshall, A. L., Str M, M. S., Bauman, A. E., Booth, M. L., Ainsworth, B. E., … Oja, P. (2003). International Physical Activity Questionnaire : 12 - Country Reliability and Validity. *Med.Sci.SportsExerc*, *35*(8), 12–1381. https://doi.org/10.1249/01.MSS.0000078924.61453.FB

Efron, B., & Tibshirani, R. J. (1994). *An Introduction to the Bootstrap*. CRC press.

Guay, F., Vallerand, R. J., & Blanchard, C. (2000). On the assessment of situational motivation scale. *Motivation and Emotion*, *24*(3), 175–213.

*Guidelines for Data Processing and Analysis of the International Physical Activity Questionnaire (IPAQ)*. (2005). Retrieved from https://sites.google.com/site/theipaq/scoring-protocol

R Core Team. (2019). *R: A language and environment for statistical computing.*

Robins, R. W., Hendin, H. M., & Trzesniewski, K. H. (2001). Measuring global self-esteem: construct validation of a single-item measure and the Rosenberg eelf-esteem scale. *Personality and Social Psychology Bulletin*, *27*(2), 151–161. https://doi.org/10.1177/0146167201272002

Rosseel, Y. (2012). **lavaan** : An *R* Package for Structural Equation Modeling. *Journal of Statistical Software*, *48*(2), 1–93. https://doi.org/10.18637/jss.v048.i02

Sallis, J. F., Pinski, R. B., Grossman, R. M., Patterson, T. L., & Nader, P. R. (1988). The development of self-efficacy scales for health-related diet and exercise behaviors. *Health Education Research*, *3*(3), 283–292. https://doi.org/10.1093/her/3.3.283

Schoemann, A. M., Boulton, A. J., & Short, S. D. (2017). Determining Power and Sample Size for Simple and Complex Mediation Models. *Social Psychological and Personality Science*, *8*(4), 379–386. https://doi.org/10.1177/1948550617715068

Stephens, T., & Craig, C. L. (1989). Fitness and Activity Measurement in the 1981 Canada Fitness Survey. In T. F. Drury (Ed.), *Assessing Physical Fitness and Physical Activity in Population-Based Surveys* (pp. 401–432). Hyattsville, MD: National Center for Health Statistics.

Tingley, D., Yamamoto, T., Hirose, K., & Keele, L. (2013). mediation : R Package for Causal Mediation Analysis, (1).

Wallston, K. a, Wallston, B. S., & Devellis, R. (1978). Development of the Multidimensional Health Locus of Control Scales. *Health Education Monographs*, *6*(2), 160–170.

Weisberg, S. (2005). *Applied Linear Regression Models* (3rd ed.). Hoboken, New Jersey: John Wiley & Sons. Retrieved from www.copyright.com.

Williams, M. N., Grajales, C. A. G., & Kurkiewicz, D. (2013). Assumptions of Multiple Regression: Correcting Two Misconceptions. *Practical Assessment, Research & Evaluation*, *18*(11). https://doi.org/10.4135/9781412950558.n433

# Appendix

# 6. Activity Adequacy Mindset Scale Validation

A validation study was conducted in order to establish internal consistency, discriminant validity, criterion validity, and test-retest reliability of the Activity Adequacy Mindset Scale (AAMS).

**6.1. Method**

**6.1.1. Participants and procedure**

**6.1.1.1. Sample S1**

Sample S1 was drawn from a research study independent of the current article. Participants were 160 community-dwelling adults (age range 19-76, mean age = 41.2; 73% female) recruited for a study on mindsets and wearable fitness technology, which allowed them to earn up to $175. Participants met the following criteria as determined via a pre-screen survey: low level of physical activity in the prior six months; walking as primary source of physical activity in the prior six months; health status allows engagement in physical activity as determined by the Physical Activity Readiness Questionnaire (PAR-Q); not pregnant (as natural changes in weight and body composition during pregnancy would invalidate results); possess an iPhone to pair with an Apple Watch. Participants attended an onboarding session on the campus of a private U.S. university. They completed a survey (including the Activity Adequacy Mindset Scale and other psychological measures), physiological health measures, and an aerobic fitness assessment.

**6.1.1.2. Sample S2**

Participants were 795 adults recruited on Amazon’s mechanical Turk (age range 18-73, mean age = 37.4; 52% female) for a study on physical activity and beliefs about health, in return for $1.60. After giving their consent, they completed a number of survey measures. Finally, 199 participants were randomly selected to be invited for a follow-up survey one week later, in order to examine test-retest reliability of AAMS; 105 participants completed this follow-up survey.

# 6.1.2. Measures

**6.1.2.1. Sample S1 Measures**

***Activity adequacy mindset.*** AAMS was measured via the same 7-item scale as described above, including the following items:

- “My current level of physical activity is healthy” (7-point scale: Strongly agree – Strongly disagree)
- “My current level of physical (in-)activity is unhealthy” (7-point scale: Strongly agree – Strongly disagree)
- “My current level of physical activity is helping me achieve or maintain a healthy body weight” (7-point scale: Strongly agree – Strongly disagree)
- How beneficial is your current level of physical activity for your health? (5-point scale: Not at all beneficial – Extremely beneficial)
- How harmful is your current level of physical activity for your health? (5-point scale: Not at all harmful – Extremely harmful)
- How much does your current level of physical (in-)activity increase or decrease your risk of disease? (7-point scale: Increases my risk very much – Decreases my risk very much)
- How much is your current level of physical (in-)activity strengthening or weakening your muscles? (7-point scale: Strengthening very much – Weakening very much)

Scale items were averaged into a composite scale after reverse-coding negatively worded items and multiplying 5-point scale items by 1.4 to ensure all items ranged from 1-7. Higher values reflect more positive mindsets.

***Other measures related to physical activity.***

*Physical activity.* Measured via the International Physical Activity Questionnaire (IPAQ), Short Last 7-Days Self-Administered Format (Craig et al., 2003), as described above (Section 3.1.3.).

*Perceived amount of exercise*. A single item asked "Overall, how much exercise did you get during the last 30 days?" (5-point scale, 1 = None at all – 5 = A great deal).

*Self-efficacy for exercise.* Measured via the same 10-item scale as described above, asking participants indicate their confidence that they can motivate themselves for at least six months to consistently do various behaviors (e.g., “Stick to your exercise program even when you have excessive demands at work/ school”; 5-point scale: 1 = Not confident at all – 5 = Extremely confident). Items were averaged into a composite.

***Other psychological measures.***

*Self-esteem.* Global self-esteem was assessed using the Single-Item Self-Esteem Scale (SISE; Robins, Hendin, & Trzesniewski, 2001)

***Health (self-reported).***

*Perceived health.* Assessed via a 1-item measure (“In general, would you say your health is…” 5-point scale: Excellent, Very Good, Good, Fair, Poor; same as above, Section 2.1.3.)

*Perceived fitness.* Assessed via a 1-item measure (“In general, how would you rate your physical fitness?” 5-point scale: 1 = Poor – 5 = Excellent)

*Physical function.* Assessed via the 4-item Physical Function subscale of the Patient-Reported Outcomes Measurement Information System (PROMIS)-29 Profile v2.0 questionnaire (e.g., “Are you able to run errands and shop?” 5-point scale, 1 = Unable to do – 5 = Without any difficulty). Items were averaged into a composite.

*Ability to participate in social roles and activities.* Assessed via the 4-item Ability to Participate in Social Roles and Activities subscale of the Patient-Reported Outcomes Measurement Information System (PROMIS)-29 Profile v2.0 questionnaire (e.g., “I have trouble doing all of my regular leisure activities with others.” 5-point scale, 1 = Always – 5 = Never). Items were averaged into a composite.

*Fatigue.* Assessed via the 4-item Fatigue subscale of the PROMIS-29 Profile v2.0 questionnaire (e.g., “In the past 30 days, I felt fatigued.” 5-point scale, 1 = Not at all – 5 = Very much). Items were averaged into a composite.

*Sleep disturbance.* Assessed via the 4-item Sleep Disturbance subscale of the PROMIS-29 Profile v2.0 questionnaire (e.g., “In the past 30 days, my sleep was refreshing.” 5-point scale, 1 = Not at all – 5 = Very much). Items were averaged into a composite.

*Anxiety.* Assessed via the 4-item Anxiety subscale of the PROMIS-29 Profile v2.0 questionnaire (e.g., “In the past 30 days, I felt fearful.” 5-point scale, 1 = Never – 5 = Always). Items were averaged into a composite.

*Depression.* Assessed via the 4-item Depression subscale of the PROMIS-29 Profile v2.0 questionnaire (e.g., “In the past 30 days, I felt hopeless.” 5-point scale, 1 = Never – 5 = Always). Items were averaged into a composite.

*Stress.* Measured via the 10-item Perceived Stress Scale (PSS; Cohen, Kamarck, & Mermelstein, 1983). Items were averaged into a composite.

***Health (physiological assessments).***

*Body mass index (BMI).* Calculated from self-reported height and weight measured on laboratory scale.

*Body fat percentage*. Measured on laboratory scale.

*Aerobic fitness.* Maximal aerobic capacity (VO2 max) assessed using the Canadian Home Fitness Test (Stephens & Craig, 1989).

**6.1.2.2. Sample S2 Measures**

***Activity adequacy mindset.*** AAMS was measured via the same 7-item scale as described above.

***Health locus of control.*** Health locus of control was assessed using the ﻿Multidimensional Health Locus of Control Scale, Form A (Wallston, Wallston, & Devellis, 1978) with 18 items including “If I get sick, it is my own behavior which determines how soon I get well again” (7-point scale: Strongly disagree – Strongly agree). Higher values denote more internal locus of control.

***Motivation for physical activity.*** The ﻿Situational Motivation Scale (SIMS; Guay, Vallerand, & Blanchard, 2000) was used to assess the extent to which participants had intrinsic, identified, external motivation or amotivation for physical activity. Participants were asked to mark the extent to which each of 16 reasons reflected why they did physical activity, including “Because I feel good when doing physical activity” (7-point scale: Not at all – Exactly).

# 6.1.3. Results

**6.1.3.1. Psychometric properties of the AAMS: Mean, SD, normality, and internal consistency**

Table S1 presents descriptive data for the AAMS. AAMS had high internal consistency, as indicated by Cronbach’s α = 0.91 in Sample S1 and Cronbach’s α = 0.94 in Sample S2.

**﻿Table S1.** Descriptive data for the Activity Adequacy Mindset Scale (AAMS).

|  | Sample S1 | Sample S2 |
| --- | --- | --- |
| *N* | 160 | 795 |
| *M* (7 items) | 3.58 | 3.74 |
| *SD* | 1.20 | 1.45 |
| Skewness | 0.26 | -0.01 |
| Kurtosis | -0.17 | -1.08 |
| Cronbach’s α | 0.91 | 0.94 |

# 6.1.3.2. Discriminant Validity

To examine whether the AAMS was distinct from other measures related to physical activity, we examined Pearson correlations between AAMS and related constructs. In Sample 1, we examined correlations with self-reported amount of physical activity (IPAQ), perceived amount of exercise, and exercise self-efficacy. We also examined whether the AAMS was distinct from global positive self-evaluation by examining its correlation with self-esteem. In Sample S2, we examined correlations with health locus of control and motivation for physical activity (intrinsic, identified, external, and amotivation).

Results are reported in Tables S2a-b. AAMS was significantly correlated in the expected direction with all measures except global self-esteem and external motivation, though these correlations were small to moderate, suggesting that the AAMS is not a redundant construct.

**﻿**

**Table S2a.** Discriminant Validity of the AAMS With Other Measures Related to Physical Activity and Self-Esteem in Sample S1 (*N* = 160).

| Variable | 1 | 2 | 3 | 4 |
| --- | --- | --- | --- | --- |
| 1. AAMS |  |  |  |  |
| 2. IPAQ | 0.35*** |  |  |  |
| 3. Perceived Exercise | 0.61*** | 0.28*** |  |  |
| 4. Exercise Self-efficacy | 0.31*** | 0.35*** | 0.34*** |  |
| 5. Self-Esteem | 0.11 | -0.01 | 0.1 | 0.064 |

*Note.* AAMS = Activity Adequacy Mindset Scale; IPAQ = International Physical Activity Questionnaire.

*** < .001; ** < .01; * < .05; ^+^ < .10

**﻿**

**Table S2b.** Discriminant Validity of the AAMS With Other Measures Related to Physical Activity in Sample S2 (*N* = 795).

| Variable | 1 | 2 | 3 | 4 | 5 |
| --- | --- | --- | --- | --- | --- |
| 1. AAMS |  |  |  |  |  |
| 2. Health Loc Control | 0.12 *** |  |  |  |  |
| 3. Intrinsic Mot | 0.57 *** | 0.14 *** |  |  |  |
| 4. Identified Mot | 0.40 *** | 0.27 *** | 0.59 *** |  |  |
| 5. External Mot | -0.03 | -0.22 *** | -0.01 | 0.11 *** |  |
| 6. Amotivation | -0.11 ** | -0.38 *** | -0.08 | -0.32 *** | 0.27 *** |

*Note.* AAMS = Activity Adequacy Mindset Scale; Health Loc Control = Health Locus of Control; Intrinsic Mot = Intrinsic Motivation for Physical Activity; Identified Mot = Identified Motivation for Physical Activity; External Mot = External Motivation for Physical Activity; Amotivation = Amotivation for Physical Activity.

*** < .001; ** < .01; * < .05; ^+^ < .10

# 6.1.3.3. Criterion Validity

﻿In order to evaluate the extent to which AAMS is related to important outcomes, Pearson correlations were computed between the AAMS and measures of health and wellbeing (collected in Sample S1). As shown in Table S3, AAMS was significantly related in the expected direction with Perceived Health, Perceived Fitness, Physical Function, Social Ability, Fatigue, Sleep Disturbance, Depression, BMI and VO2 max. The correlation with Stress was trending (*p* = .076), and the correlation with Anxiety was non-significant.

**Table S3.** Criterion Validity of the AAMS With Measures of Health and Wellbeing in Sample S1 (*N* = 160).

| Variable | 1 | 2 | 3 | 4 | 5 | 6 | 7 | 8 | 9 | 10 | 11 |
| --- | --- | --- | --- | --- | --- | --- | --- | --- | --- | --- | --- |
| 1. AAMS |  |  |  |  |  |  |  |  |  |  |  |
| 2. Perc Health | .50*** |  |  |  |  |  |  |  |  |  |  |
| 3. Perc Fitness | .58*** | .70*** |  |  |  |  |  |  |  |  |  |
| 4. Phys Funct | .18* | .19* | .13 |  |  |  |  |  |  |  |  |
| 5. Social Abl | .23** | .17* | .10 | .50*** |  |  |  |  |  |  |  |
| 6. Fatigue | -.26*** | -.32*** | -.27*** | -.25** | -.39*** |  |  |  |  |  |  |
| 7. Sleep Dis | -.21** | -.31*** | -.24** | -.18* | -.30*** | .54*** |  |  |  |  |  |
| 8. Anxiety | -.13 | -.23** | -.14^+^ | -.24** | -.39*** | .47*** | .32*** |  |  |  |  |
| 9. Depression | -.16* | -.32*** | -.18* | -.24** | -.39*** | .46*** | .36*** | .80*** |  |  |  |
| 10. Stress | -.14^+^ | -.22** | -.19* | -.10 | -.33*** | .48*** | .37*** | .76*** | .69*** |  |  |
| 11. BMI | -.28*** | -.31*** | -.32*** | -.21** | -.09 | .20* | .14^+^ | .16^+^ | .20* | .10 |  |
| 12. VO2max | .27*** | -.04 | .16* | .22** | .12 | -.04 | .07 | .02 | .02 | .15^+^ | -.41*** |

*Note.* AAMS = Activity Adequacy Mindset Scale; Perc Health = Perceived Health; Perc Fitness = Perceived Fitness; Phys Funct = Physical Functioning; Social Abl = Ability to Participate in Social Roles and Activities; Sleep Dis = Sleep Disturbance.

*** < .001; ** < .01; * < .05; ^+^ < .10

Additionally, to examine whether AAMS predicts unique variance in health and wellbeing outcomes, hierarchical linear regression was conducted predicting each outcome variable with amount of physical activity (IPAQ), perceived exercise, and exercise self-efficacy entered as predictors in Step 1, and AAMS entered in Step 2.

AAMS was a significant predictor of unique variance over and above the other predictors in Perceived Health (β = 0.34, *t*(155) = 3.824, *p* < .001; Δ*R*^2^ = 0.07), Perceived Fitness (β = 0.36, *t*(155) = 4.484, *p* < .001; Δ*R*^2^ = 0.08), Ability to Participate in Social Roles and Activities (β = 0.25, *t*(155) = 2.426, *p* = .016; Δ*R*^2^ = 0.04), Fatigue (β = 0.24, *t*(155) = 2.375, *p* = .019; Δ*R*^2^ = 0.03), Sleep Disturbance (β = 0.21, *t*(155) = 2.036, *p* = .044; Δ*R*^2^ = 0.03), BMI (β = -0.30, *t*(154) = -3.008, *p* = .003; Δ*R*^2^ = 0.05), and VO2 max (β = 0.22, *t*(153) = 2.180, *p* = .031; Δ*R*^2^ = 0.03).

AAMS was a marginally significant predictor of unique variance over and above the other predictors in Stress (β = 0.18, *t*(155) = 1.734, *p* = .085; Δ*R*^2^ = 0.02) and Depression (β = 0.19, *t*(155) = 1.807, *p* = .073; Δ*R*^2^ = 0.2).

AAMS did not significantly predict unique variance over and above the other predictors in Physical Function (β = 0.14, *t*(155) = 1.396, *p* = .165; Δ*R*^2^ = 0.01) or Anxiety (β = 0.16, *t*(155) = 1.587, *p* = .115; Δ*R*^2^ = 0.02).

# 6.1.3.4. Test-Retest Reliability

To examine test-retest reliability, Pearson correlations were computed between AAMS scores (average of 7 AAMS items) at Time 1 and Time 2 (assessed one week later; Sample S2). The correlation was r = 0.83, indicating good test-retest reliability.

# 7. Pre-Registration Document (Also available at https://aspredicted.org/bh3st.pdf)
